# Alterations in micro RNA-messenger RNA (miRNA-mRNA) Coupled Signaling Networks in Sporadic Alzheimer’s Disease (AD) Hippocampal CA1

**DOI:** 10.4172/2161-0460.1000312

**Published:** 2017-03-10

**Authors:** V Jaber, Y Zhao, WJ Lukiw

**Affiliations:** 1 LSU Neuroscience Center, Louisiana State University Health Sciences Center, 2020 Gravier Street, Suite 904, New Orleans LA 70112 USA; 2 Department of Cell Biology and Anatomy, Louisiana State University Health Sciences Center, 2020 Gravier Street, Suite 904, New Orleans LA 70112 USA; 3 Departments of Ophthalmology and Neurology, Louisiana State University Health Sciences Center, 2020 Gravier Street, Suite 904, New Orleans LA 70112 USA

**Keywords:** Bioinformatics, DNA microfluidic array, Inflammation, messenger RNA (mRNA), microRNA (miRNA), miRNA-34a, miRNA-146a, Phagocytosis, Synaptogenesis

## Abstract

RNA sequencing, DNA microfluidic array, LED-Northern, Western immunoassay and bioinformatics analysis have uncovered a small family of up-regulated human brain enriched microRNAs (miRNAs) and down-regulated messenger RNAs (mRNAs) in short post-mortem interval (PMI) sporadic Alzheimer’s disease (AD) brain. At the mRNA level, a large majority of the expression of human brain genes found to be down-regulated in sporadic AD appears to be a consequence of an up-regulation of a specific group of NF-kB-inducible microRNAs (miRNAs). This group of up-regulated miRNAs – including miRNA-34a and miRNA-146a - has strong, energetically favorable, complimentary RNA sequences in the 3′ untranslated regions (3′-UTR) of their target mRNAs which ultimately drive the down-regulation in the expression of certain essential brain genes. Interestingly, just 2 significantly up-regulated miRNAs - miRNA-34a and miRNA-146a – appear to down-regulate mRNA targets involved in synaptogenesis (SHANK3), phagocytosis deficits and tau pathology (TREM2), inflammation (CFH; complement factor H) and amyloidogenesis (TSPAN12), all of which are distinguishing pathological features characteristic of middle-to-late stage AD neuropathology. This paper reports the novel finding of parallel miRNA-34a and miRNA-146a up-regulation in sporadic AD hippocampal CA1 RNA pools and proposes an altered miRNA-mRNA coupled signaling network in AD, much of which is supported by current experimental findings in the recent literature.

## Overview

**Figure 1:**
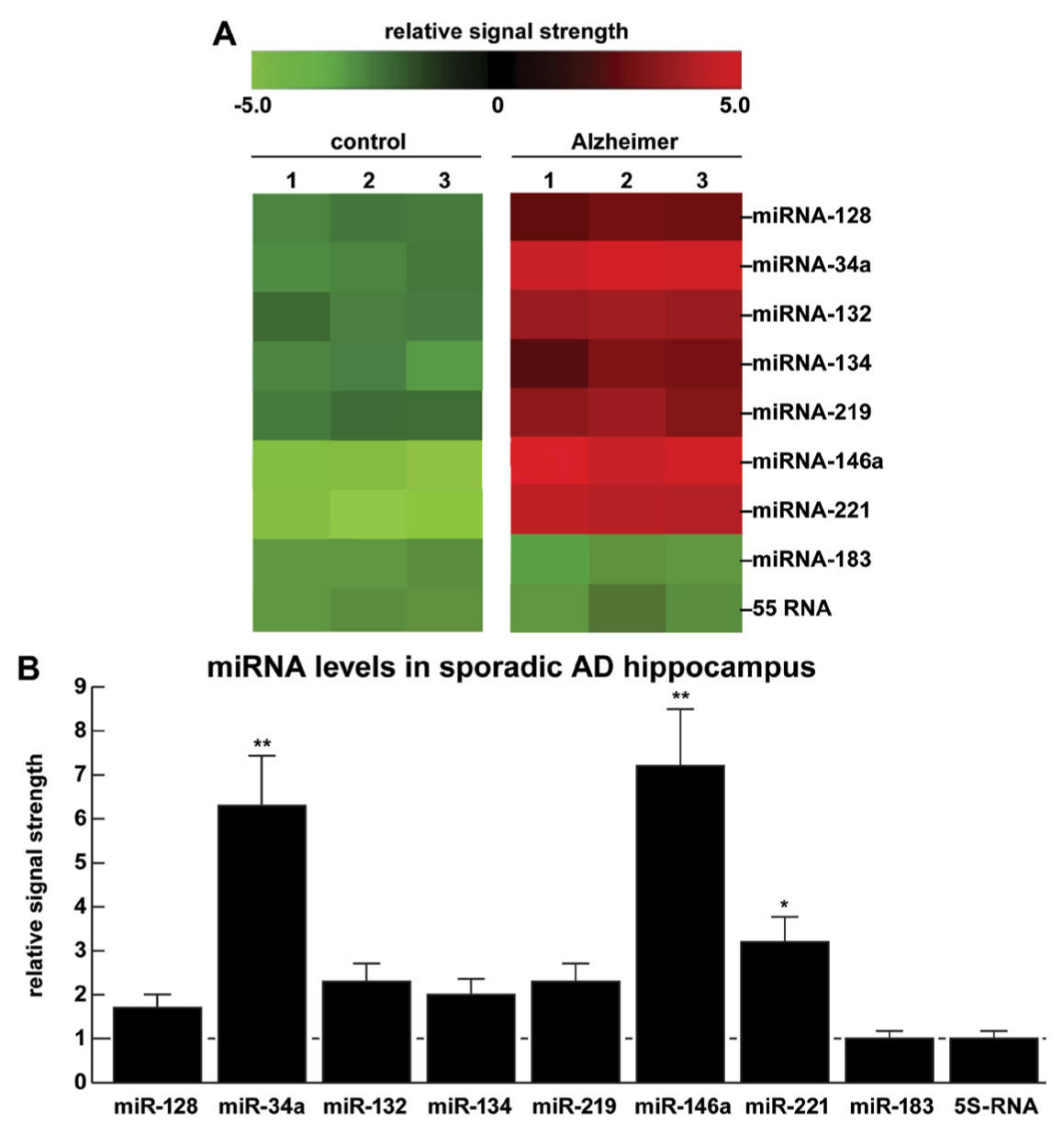
(A) Color-coded cluster analysis of a small family of up-regulated miRNA species in the hippocampal CA1 region of 9 pooled control and 9 pooled moderate-to-late-stage AD cases; [experiments performed in triplicate (1–3) age range for AD and control cases 54–72 year; all females; controls mean +/−1 SD=63.0+/−8.9 year; AD mean +/−1 SD=64.8+/−10.2 year]; miRNA levels analyzed in triplicate [1–3] using RNA sequencing, LED Northern and/or microfluidic miRNA arrays (LC Sciences, Houston TX) [1,4]; these up-regulated miRNAs include, prominently, miRNA-34a and miRNA-146a, two pathogenic miRNAs known to be linked to the innate-immune response, the propagation of inflammatory neurodegeneration, altered synapto-genesis and amyloidogenesis [10–13]; analogous studies in mild cognitive impairment (MCI; a potential ‘precursor’ to moderate-to-late-stage AD) tissues may be further useful in expanding our understanding of the evolution and establishment of miRNA-mRNA coupled signaling networks; control miRNA-183 and the small structural 5S RNA did not change under these conditions and were used as internal control markers in the same sample; (B) quantification of data in bar graph format; interestingly miRNA-34a and miRNA-146a (established markers for human pro-inflammatory neurodegeneration) are also seen to be significantly up-regulated in other neuropsychiatric disorders including autism spectrum disorder (ASD) and prion disease [12–21; unpublished]; we have additional evidence that up-regulated miRNA-34a and miRNA-146a down-regulate expression of SHANK3, a scaffold protein essential for synapse formation and communication between neurons, and a contributor to the AD phenotype and related neuropsychiatric disorders (Figures 2 and 3; 21,22; unpublished); in these experiments there was no statistical difference between control and AD mean age, gender, PMI or RNA quality; in (B) horizontal line at 1.0 for ease of comparison against controls; N=3; *p<0.05; **p<0.01 (ANOVA).

**Figure 2:**
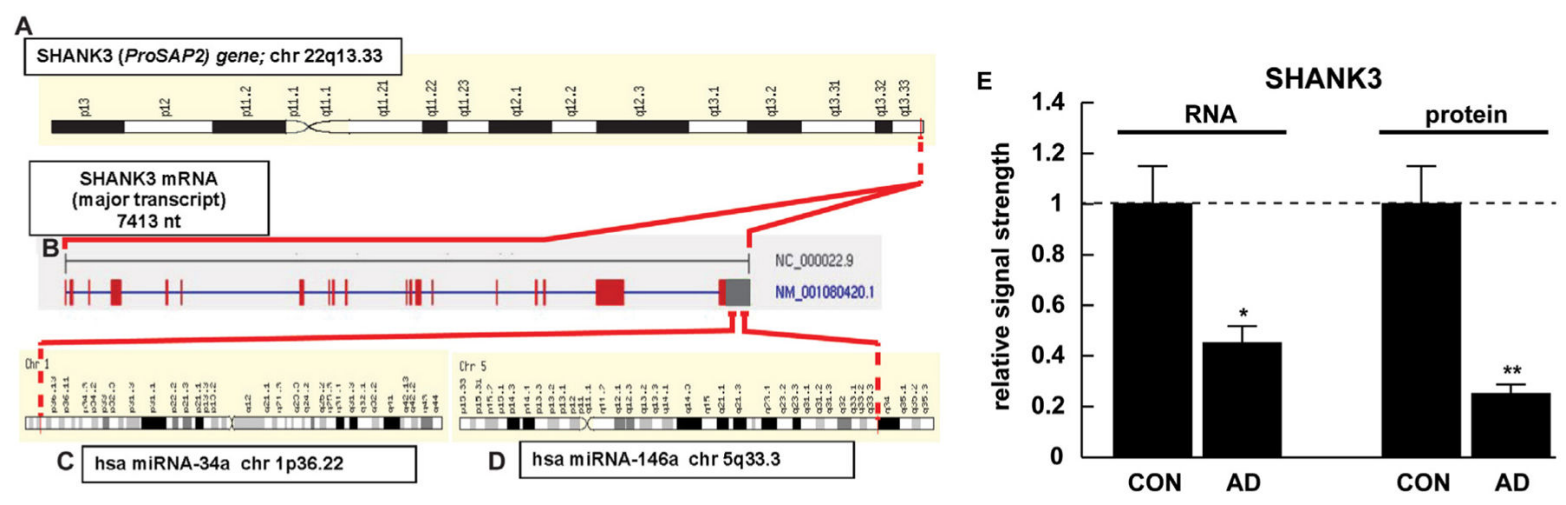
Structure of the human SHANK3 (ProSAP2) gene (chr22q13.33), homo sapien (hsa) miRNA-34a (chr 1p36.22) and miRNA-146a (chr 5q33.3) genes; SHANK3 is a multidomain synapse and scaffolding protein whose deficits are strongly linked to the neurodegenerative phenotype; (A) the SHANK3 gene is encoded on the distal arm of chr 22 encoding (B) a major 22 exon (red boxes) 7413 nucleotide (nt) SHANK3 mRNA (NM_ 001080420.1); in the regulation of SHANK3 gene expression the SHANK3 mRNA 3′-UTR [gray box at the right end of (B) NM_ 001080420.1] is targeted by two strongly pro-inflammatory, inducible microRNAs (C) miRNA-34a and (D) miRNA-146a; both SHANK3 mRNA 3′-UTR-miRNA-34a and -miRNA-146a have very strong bonding energies of −22 kcal/mol or less (unpublished); transfection/luciferase reporter assays using pLightSwitch vectors also show that miRNA-34a and miRNA-146a have very strong affinities for the SHANK3 mRNA 3′-UTR (unpublished; manuscript in preparation); in the same tissues from which total mRNA and total miRNA was extracted, SHANK3 mRNA was found to be decreased to 0.45-fold of control, and SHANK3 protein was found to be decreased to 0.27-fold of control; p*<0.05, p**<0.01 (ANOVA); deficits in SHANK3 expression in AD hippocampal CA1 might be expected to contribute to synaptic deficits as are observed in AD (Figure 3); in (E) a horizontal line at 1.0 (corresponding to controls) is shown for ease of comparison against AD cases; N=3; *p<0.05; **p<0.01 (ANOVA).

**Figure 3:**
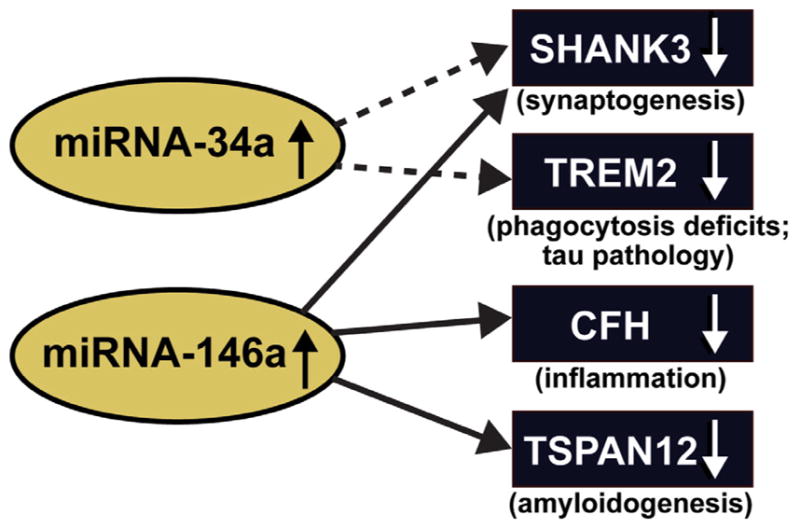
Potential pathways to the acquisition of the AD phenotype via multiple miRNA-mRNA interactions; AD brains are associated with non-random increases in the abundance of select pro-inflammatory pathogenic miRNAs including miRNA-34a (dashed arrows) and miRNA-146a (solid arrows); the reason for this selective increase is not well understood but it is known that both miRNA-34a and miRNA-146a are both inducible by the pro-inflammatory transcription factor NF-kB [23–25]; increases in miRNA-34a and miRNA-146a are known to be associated with brain inflammation, changes and/or deficits in the innate-immune system and phagocytosis, tau pathology, synaptic aberrations and deficits in synaptogenesis; via miRNA-mRNA 3′-UTR interactions (Figure 2) up-regulated miRNA-34a and miRNA-146a target strongly down-regulate the expression of SHANK3, a multidomain synapse and scaffolding protein whose deficits are strongly linked to the acquisition of the ‘inflammatory neurodegeneration’ phenotype as exemplified by AD in the brain and age-related macular degeneration (AMD) in the retina; there is a predicted interplay between the down-regulation of SHANK3 gene expression and alterations in inflammation, the innate-immune response and synaptic aberrations which are not well understood; major consequences of up-regulated miRNA-34a and miRNA-146a include (i) the promotion of inflammation and induced changes in the innate-immune signalling [14–20]; and (ii) the down-regulation of the synapse-associated protein SHANK3 and synaptic aberrations leading to synaptic signalling dysfunction as is observed in AD brain [1,24,25].

microRNAs (miRNAs) are typically ~21–23 nucleotide (nt) non-coding, single stranded RNAs (ssRNAs) that have established themselves as significant post-transcriptional regulators of messenger RNA (mRNA) abundance, speciation and complexity in the human central nervous system (CNS). In various tissues and compartments of the CNS these RNA signaling molecules appear to play important roles in development, aging, health and disease [1–4]. To date about 2650 different miRNAs have been identified in all human tissues; only about 35–40 miRNAs are relatively abundant in the human brain, underscoring the high selection pressure carried by a highly select group of specific ribonucleotide sequences that in part characterize these highly mobile and soluble ssRNAs [2–10]. Major schemes of miRNA action on their specific mRNA targets include several noteworthy observations: (i) that up-regulated mature miRNAs generally interact, via ribonucleotide base pair complementarity, with the 3′-untranslated region (3′-UTR) of their target mRNA(s) and this results in the down-regulation or elimination of expression of that mRNA [5,6,10]; (ii) that one miRNA can target multiple mRNA 3′-UTRs and conversely, multiple miRNAs can target a single mRNA 3′-UTR (Figures 1 and 2) [5–8]; (iii) that up-regulated miRNAs for the most part down-regulate their mRNA targets via a ‘productive’ mRNA 3′-UTR recognition and interaction [4,5,8]; (iv) that miRNAs can act both as an ‘on-off switch’ or as ‘a fine-tuner’ of mRNA-driven gene expression via ‘threshold effects’ [5,8,9]; (v) that miRNAs and their target mRNAs are more often than not transcribed from separate chromosomes - thereby gene transcription products originating from at least 2 different and unlinked RNA Pol II transcribed chromosomes may be needed to orchestrate the expression of specific miRNA-regulated mRNAs [6–8] (Figure 2); (vi) that only about one sixth of the 35–40 miRNAs that are abundantly expressed in the human CNS appear to be significantly up-regulated in the AD brain and in stressed human brain cells in primary culture (see below) [9,10]; (vii) that chronic inflammatory neurodegenerative diseases of the brain and retina such as AD and age-related macular degeneration (AMD) as well as inflammatory aspects of epilepsy, multiple sclerosis and prion disease also share an up-regulation of many of these same pro-inflammatory miRNAs [11–20]; (viii) that in stressed primary human brain cell culture experiments these same miRNAs found to be up-regulated in sporadic AD (miRNA-34a and miRNA-146a) were found to be under transcriptional regulatory control by the pro-inflammatory transcription factor NF-kB, and were inducible from outside the cell [18–21]; and (ix) that the up-regulated miRNAs that characterize AD and chronic age-related inflammatory degeneration of the brain are strongly associated with deficits in the homeostatic expression of brain gene families involved in amyloidogenesis, inflammation, the innate-immune response, neurotrophism and synaptogenesis [7,10–21] (Figure 3). While other miRNA and mRNA circuits may be additionally involved, we find it intriguing (i) that a select family of just 2 inducible, hippocampal CA1-enriched miRNAs and their interactions with multiple mRNA targets in brain cells can explain much of the observed neuropathological characteristics of sporadic AD; and (ii) that their altered abundance is comparable to those found in other chronic, progressive inflammatory neurodegenerations of the human CNS that include epilepsy, multiple sclerosis and prion disease [11–20] (Figures 1–3).

## Generalized down-regulation of gene expression in sporadic AD via miRNA up-regulation

Early studies on global gene expression patterns in the human brain neocortex and hippocampus using Northern, RT-PCR and/or DNA array analytical approaches indicated that sporadic AD brain tissues were characterized by a general and significant down-regulation in gene expression of brain-essential components including neuron-specific, neurotropic and synaptogenic markers, and this was linked to an up-regulation of pro-inflammatory, amyloidogenic and altered synaptic signaling [1–3,12,16]. The mechanism of increased miRNA abundance, due to increased NF-kB activation and signaling in their respective promoters, followed by subsequent decreases in their target mRNAs and hence decreases in the expression from those target mRNAs provided an acceptable explanation for these highly interactive pathogenetic events involving altered gene expression patterns [1–5,20] (Figures 1–3). Interestingly, the up-regulation of a small family of inducible miRNAs such as miRNA-34a and miRNA-146a and others in both human brain cell co-cultures and AD brain tissues when compared to age-matched controls in part has defined a network of pathogenic miRNA-mRNA coupled signaling [4,6–15] (Figure 3). What are particularly attractive in this pathogenetic network is the kinds of mRNAs that are being down-regulated by these up-regulated miRNAs which can explain much of the neuropathology – progressive amyloidogenesis, inflammation and defective neurotrophism, altered innate-immune signaling and impaired synaptogenesis characteristic of the sporadic AD process.

## Recent advances-down-regulation in the expression of SHANK3, TREM2, CFH AND TSPAN12

The expression of certain key mRNAs appears to be significantly down-regulated in sporadic AD brain. Recently these have included SHANK3 [22], TREM2 [11,12,25], CFH [7] and TSPAN12 [13,21] (Figure 3); and each of these brain-abundant proteins have been shown to play critical roles in the altered neurobiology and neuropathology that characterize the AD brain. For example, expression of the CNS-enriched SHANK3 gene that encodes core scaffolding proteins at the glutamatergic synapse is a critical regulator of synaptic form and function that is altered in AD brain, and appears to play a direct role in altered glutamatergic neurotransmission [22]. Down-regulation of the triggering receptor enriched in myeloid/microglial cells (TREM2), a transmembrane glycoprotein enriched in the brain’s microglial ‘immune surveillance’ cells similarly appears to play a role in tau pathology and the inability of brain cells in AD to efficiently phagocytose pathological proteins that include amyloid peptides [11,12,25] (Figure 3). Similarly, down-regulation of complement factor H (CFH) expression appears to increase innate-immune signaling with significant ectopic activation of the complement system, with pro-inflammatory consequences [7]. Analogously, insufficiency of the signal transducing tetraspanin12 (TSPAN12) protein that resides adjacent to the membrane-spanning beta amyloid precursor protein (βAPP) complex has been shown to direct enhanced Aβ42 peptide generation from the βAPP precursor [13,21]. Interestingly, (i) each of these 4 AD-relevant biomarkers (SHANK3, TREM2, CFH and TSPAN12) have recently been shown to be significantly down-regulated via up-regulation of inducible miRNA-34a and/or miRNA-146a signals in AD brain and/or in AD models (Figure 3); (ii) mutations in most of these genes in familial AD lead to a loss-of-function - so loss-of-function of a specific gene expression product (such as SHANK3, TREM2, CFH or TSPAN12) may have the equivalent physiological effect as not having enough of a normally functioning gene product; and (iii) overlapping high affinity miRNA-mRNA 3′-UTR binding sites, such as the overlapping miRNA-146a and miRNA-155 in the 232 nucleotide CFH mRNA 3′-UTR (energy of association of less than −22 kcal/mol; Genbank BC142688) may add a further layer of complexity to the regulation of brain - and/or retina-enriched gene expression when multiple miRNAs target the same mRNA 3′-UTR [6,7].

## Pooling of human brain samples for gene expression analysis

Through the pooling of control and AD tissue samples from the same anatomical region and matched for age, gender and post-mortem interval (PMI) allows for the identification of “general trends” in gene expression patterns for that anatomical region (Figure 1) [1–4,16,20]. For example, pooling of controls in one group and AD samples in another group from the same anatomical region and matched as closely as possible for age, gender, and PMI, it was recently possible to show (i) significant reductions in AD in the expression of the triggering receptor expressed in myeloid/microglial cells (TREM2), a microglial-resident sensor-receptor glycoprotein important in the clearance of amyloid peptides from the brain’s extracellular space [11,12]; and (ii) statistically significant individual differences in abundance and complexity of a subfamily of pathology-associated and AD-relevant miRNAs between Caucasian and African-American populations. Using gene expression analysis of short PMI tissues, it is most important that an accurate diagnosis of a neurological disorder be made and what other pathophysiological, epigenetic, microbial, dietary, lifestyle or environmental disturbances may have contributed to the disease phenotype. This may be particularly critical in the diagnosis of AD where extensive neuropathological examination of post-mortem brain, and especially the quantitation of senile plaque and neurofibrillary tangle densities and other inflammation-related biomarkers, is still considered by many to be the “gold standard” for the most accurate diagnosis of AD [12,16]. Lastly, it is important to point out that besides the rigorous quality control essential for meaningful miRNA and mRNA analysis, the integration of post-mortem neuropathological examination with pre-mortem pathological factors including familial history, lifestyle factors, drug history, neuroimaging data, and serum and CSF profiles will continue to contribute to the discovery of altered gene expression patterns and novel biomarker discovery, the advancement of AD diagnostics, and ongoing analytical standardization [10,11,16,27–30].

## An integrated miRNA-mRNA profile and altered gene expression network in sporadic AD

It is important to point out: (i) that the levels of miRNA and mRNA in pathological tissues compared to age matched controls in the same anatomical region is at best a ‘*physiological snapshot*’ of what is going on inside that cell precisely at the time analyzed; (ii) the remarkable redundancy of gene expression in cells and tissues with high transcription rates [1,28,30]; and that these gene expression profiles can represent a somewhat skewed portrait of ‘*a highly dynamic neurogenetic’* process that is both constantly changing, and continuously under the powerful influence of many other genetic, epigenetic and environmental factors [27–30]. Another major confounding issue is that the genetics, epigenetics and environmental effects varies widely amongst different human populations with different genetic backgrounds, and these observations are in accordance with the concept of “*human genetic individuality*” in which there are real and significant inter-ethnic differences in sporadic AD epidemiology, incidence, disease course and progression [29,30]. On the other hand the observation that certain species of miRNA and mRNA are commonly altered in abundance in control and AD brain tissues from different sources may underscore the importance of these mis-regulated RNA species, their altered abundance, speciation and complexity and how they contribute to the neuropathology of the AD process.

## Summary

AD remains a rapidly expanding health and socioeconomic concern in industrialized societies and the leading cause of intellectual impairment and progressive dementia in our aging population. Indeed, few fatal neurological disorders of the human CNS present with the complexity, heterogeneity and insidiousness of sporadic AD. Susceptibility genes that have been identified for AD are involved in an exceptionally wide and varied range of basic neurobiological functions, however their generalized down-regulation in expression contribute to altered synaptogenesis, deficits in phagocytosis, increased inflammation and/or amyloidogenesis that together characterize major pathological features of AD (Figure 3) [27–30]. Elucidation of the sporadic AD mechanism and genetic and epigenetic factors that contribute to the initiation, progression, and spreading of this chronic and fatal neurodegeneration will ultimately result in improved and effective diagnostics and intervention strategies. The relatively recent discovery of up-regulated, inducible, NF-kB-regulated miRNAs in the human CNS and altered miRNA-mRNA coupled networks in AD brain is revealing an entirely new layer of post-transcriptional gene control in sporadic AD – and together this has greatly expanded our perception, knowledge and understanding of the complexity and dynamics of pathogenic gene regulation in the human CNS [25–30]. Indeed up-regulated miRNA-34a and miRNA-146a and their down-regulated mRNA and gene expression targets that include SHANK3, TREM2, CFH and TSPAN12 may be part of a much larger and dynamic miRNA-mRNA coupled signaling system operating in the AD brain. Even though the exact cause of sporadic AD remains elusive, and there are currently no effective treatments to stop or reverse the progression of this uniquely human and age-related neurological disorder, anti-NF-kB and/or anti-miRNA strategies pose a realistically exploitable and readily available therapeutic approach for intervention of progressive degenerative processes in the CNS that may be genuinely useful in the clinical management of AD.

## References

[1] Colangelo V, Schurr J, Ball MJ, Pelaez RP, Bazan NG (2002). Gene expression profiling of 12633 genes in Alzheimer hippocampal CA: Transcription and neurotrophic factor down-regulation and up-regulation of apoptotic and pro-inflammatory signaling. J Neurosci Res.

[2] Mufson EJ, Counts SE, Che S, Ginsberg SD (2006). Neuronal gene expression profiling: Uncovering the molecular biology of neurodegenerative disease. Prog Brain Res.

[3] Courtney E, Kornfeld S, Janitz K, Janitz M (2010). Transcriptome profiling in neurodegenerative disease. J Neurosci Methods.

[4] Zhao Y, Alexandrov PN, Jaber V, Lukiw WJ (2016). Deficiency in the ubiquitin conjugating enzyme UBE2A in Alzheimer’s disease (AD) is linked to deficits in a natural circular miRNA-7 sponge (circRNA; ciRS-7). Genes (Basel).

[5] Guo H, Ingolia NT, Weissman JS, Bartel DP (2010). Mammalian microRNAs predominantly act to decrease target mRNA levels. Nature.

[6] Lukiw WJ, Alexandrov PN, Zhao Y, Hill JM, Bhattacharjee S (2012). Spreading of Alzheimer’s disease inflammatory signaling through soluble micro-RNA. Neuroreport.

[7] Lukiw WJ, Surjyadipta B, Dua P, Alexandrov PN (2012). Common micro RNAs (miRNAs) target complement factor H (CFH) regulation in Alzheimer’s disease (AD) and in age-related macular degeneration (AMD). Int J Biochem Mol Biol.

[8] Woldemichael BT, Mansuy IM (2016). Micro-RNAs in cognition and cognitive disorders: Potential for novel biomarkers and therapeutics. Biochem Pharmacol.

[9] Cipolla GA (2014). A non-canonical landscape of the microRNA system. Front Genet.

[10] Hill JM, Lukiw WJ (2016). MicroRNA (miRNA)-mediated pathogenetic signaling in Alzheimer’s disease (AD). Neurochem Res.

[11] Bhattacharjee S, Zhao Y, Dua P, Rogaev EI, Lukiw WJ (2016). microRNA-34a-mediated down-regulation of the microglial-enriched triggering receptor and phagocytosis-sensor TREM2 in age-related macular degeneration. PLoS One.

[12] Zhao Y, Jaber V, Lukiw WJ (2016). Over-expressed pathogenic miRNAs in Alzheimer’s disease (AD) and prion disease (PrD) drive deficits in TREM2-mediated Aβ42 peptide clearance. Front Aging Neurosci.

[13] Li YY, Cui JG, Dua P, Pogue AI, Bhattacharjee S (2011). Differential expression of miRNA-146a-regulated inflammatory genes in human primary neural, astroglial and microglial cells. Neurosci Lett.

[14] Lukiw WJ, Zhao Y, Cui JG (2008). An NF-kB-sensitive micro RNA-146a-mediated inflammatory circuit in Alzheimer disease and in stressed human brain cells. J Biol Chem.

[15] Saba R, Medina SJ, Booth SA (2014). A functional SNP catalog of overlapping miRNA-binding sites in genes implicated in prion disease and other neurodegenerative disorders. Hum Mutat.

[16] Clement C, Hill JM, Dua P, Culicchia F (2016). Analysis of RNA from Alzheimer’s disease post-mortem brain tissues. Mol Neurobiol.

[17] Zhao Y, Bhattacharjee S, Jones BM, Hill JM, Clement C (2015). Beta-amyloid precursor protein (βAPP) processing in Alzheimer’s disease (AD) and age-related macular degeneration (AMD). Mol Neurobiol.

[18] Aronica E, Fluiter K, Iyer A, Zurolo E, Vreijling J (2010). Expression pattern of miRNA-146a, an inflammation-associated microRNA, in experimental and human temporal lobe epilepsy. Eur J Neurosci.

[19] Devier DJ, Lovera JF, Lukiw WJ (2015). Increase in NF-κB-sensitive miRNA-146a andmiRNA-155 in multiple sclerosis (MS) and pro-inflammatory neurodegeneration. Front Mol Neurosci.

[20] Zhao Y, Bhattacharjee S, Dua P, Alexandrov PN, Lukiw WJ (2015). microRNA-based biomarkers and the diagnosis of Alzheimer’s disease. Front Neurol.

[21] Lukiw WJ (2012). NF-κB-regulated micro RNAs (miRNAs) in primary human brain cells. Exp Neurol.

[22] Choi SY, Pang K, Kim JY, Ryu JR, Kang H (2015). Post-transcriptional regulation of SHANK3 expression by microRNAs related to multiple neuropsychiatric disorders. Mol Brain.

[23] Guilmatre A, Huguet G, Delorme R, Bourgeron T (2014). The emerging role of SHANK genes in neuropsychiatric disorders. Dev Neurobiol.

[24] Monteiro P, Feng G (2017). SHANK proteins: roles at the synapse and in autism spectrum disorder. Nat Rev Neurosci.

[25] Zhao Y, Bhattacharjee S, Jones BM, Dua P, Alexandrov PN (2013). Regulation of TREM2 expression by an NF-κB-sensitive miRNA-34a. Neuroreport.

[26] Snow WM, Albensi BC (2016). Neuronal gene targets of NF-κB and their dysregulation in Alzheimer’s disease. Front Mol Neurosci.

[27] Jang SS, Chung HJ (2016). Emerging link between Alzheimer’s disease and homeostatic synaptic plasticity. Neural Plast.

[28] Skaper SD, Facci L, Zusso M, Giusti P (2017). Synaptic plasticity, dementia and Alzheimer disease. CNS Neurol Disord Drug Targets.

[29] Lukiw WJ (2013). Variability in micro RNA (miRNA) abundance, speciation and complexity amongst different human populations and potential relevance to Alzheimer’s disease (AD). Front Cell Neurosci.

[30] Sims R, Williams J (2016). Defining the genetic architecture of Alzheimer’s disease: Where next. Neurodegener Dis.

